# Effect of Removal of Enamel on Rebonding Strength of Resin Composite to Enamel

**DOI:** 10.1155/2016/1818939

**Published:** 2016-09-20

**Authors:** L. Kilponen, L. Lassila, M. Tolvanen, J. Varrela, P. K. Vallittu

**Affiliations:** ^1^Institute of Dentistry, University of Turku, Turku, Finland; ^2^Turku Clinical Biomaterials Centre (TCBC), Department of Biomaterials Science, Institute of Dentistry, University of Turku, Turku, Finland; ^3^Department of Community Dentistry, Institute of Dentistry, University of Turku, Turku, Finland; ^4^Department of Oral Development and Orthodontics, Institute of Dentistry, University of Turku, Turku, Finland; ^5^City of Turku, Division of Welfare, Turku, Finland

## Abstract

*Objective*. To examine the effect of removing the surface layer of enamel on the rebonding strength of resin composite.* Methods*. Teeth in four groups (*n* = 10) were etched, a small amount of resin composite was bonded and debonded, then specimens in three groups were ground for different lengths of time (10 s, 20 s, 30 s) to remove an increasing amount of enamel, one group was left untouched. The teeth were bonded again and the bond strengths of 1st and 2nd bonding were compared and analysed against the amount of enamel loss in different groups (7 *µ*m (±2); 12 *µ*m (±1); 16 *µ*m (±3)). Specimens were examined with SEM and by noncontacting optical profilometer.* Results*. Although results indicated higher rebonding strength with increasing enamel removal ANOVA showed low statistical differences between the groups (*p* > 0.05). However, values between first bonding and rebonding strengths differed significantly (*p* < 0.05) in the group that was not ground. SEM revealed that enamel-surfaces that were ground after debonding etched well, compared to the surfaces that still contained adhesive remnants.* Conclusions*. Removal of small amount of enamel refreshed the surface for rebonding. Rebonding strengths without grinding the surface before bonding were lower than bond strength to intact enamel.

## 1. Introduction

The etching of enamel is the basis of bonding resins and resin composites to enamel, as it increases the surface area and the surface energy of enamel [1]. Etching produces microporosities into which the adhesive can flow and form a structure of tags, and this micromechanical retention leads to a stronger bond [2, 3]. The depth of the resin tag formation is reported to vary between 5 and 50 *µ*m [3–6]. It has been demonstrated that increasing the length of the tags contributes little to the bond strength [5]. Etching produces a preferential etching pattern depending on the direction of the enamel rods. The two most common patterns are type one where the center of the prisms is dissolved and type two where the prism periphery is dissolved. A third pattern also exists where no prism structure is evident [7–9].

Phosphoric acid etching with an acid concentration of 10–30% produces the highest bond strengths [10]. These acid concentrations generate maximum enamel dissolution and deposits removable by thorough water-rinsing. In clinical work, concentrations of 30–40% are commonly used. Bond strengths measured by so-called shear bond method to etched enamel with different etching times are as an average 20 MPa, which is considered sufficient and represents the highest bonding values which are available with dental tissues and resin composites [11, 12]. A large variety of dental restorative and active orthodontic treatments are based on enamel bonding. However, sometimes debondings occur and there is need for rebonding of the restoration or orthodontic device. Typically, some amount of enamel is removed for the rebonding procedure.

There can be a difference in the generated etching pattern depending on whether the enamel is intact or instrumented, for example, by grinding. In mature enamel, there is a mineralization gradient, increasing from inner to outer enamel, and there can also be differences in the composition of enamel due to patient's age, properties of saliva, fluoride concentration of drinking water, and so forth. In a young intact tooth, the outermost layer of enamel is aprismatic, that is, more condensed. Also, salivary calcium ions can mineralize the enamel and fluorine ions can transform the hydroxyapatite to fluoroapatite. Therefore the superficial enamel layer is usually harder than the inner enamel [9, 13, 14]. The etching result of intact enamel depends on the characteristics of the specific area of enamel and may not be uniform over the whole enamel area [15, 16]. In some studies, it has been reported that when etching with 32–35% phosphoric acid, the intact and ground or otherwise instrumented enamel surfaces both develop a porous surface and the bond strength is similar for both surfaces [17].

When rebonding orthodontic brackets, or when recementing loose adhesive restorations, the properties of the underlying layer of previously treated enamel can affect the rebonding strength. The surface of the enamel can contain adhesive remains even after removing all visible adhesive with a scaler [18]. The remaining adhesive can decrease the roughness of the enamel surface and therefore diminish also the rebonding strength [19, 20]. On the other hand, it has also been suggested that the residual adhesive provides a surface for the new adhesive to bond to, chemically or mechanically [21]. It has been reported that reetching does not remove this residual adhesive [19, 20], and therefore a method that does remove the surface enamel should be employed. The bond strength values in rebonding can be inconsistent. Some studies have found the rebonding strength to be lower than the initial bond strength [19, 20, 22, 23], whereas other studies have found the rebonding strength to be higher than the initial bond strength and attribute this to, for example, an increase in enamel roughness caused by the residual adhesive removal [24].

The objective of this study was to examine systematically the effect of removing the surface layer of enamel in rebonding procedures and to analyse microstructural changes at the teeth surfaces.

## 2. Materials and Methods

The teeth used in the study were extracted molars acquired from the teaching clinic of Institute of Dentistry, University of Turku, Turku, Finland. The roots of the teeth were cut off with a histological saw (Secotom-50, Struers A/S, Ballerup, Denmark) and 40 teeth were horizontally embedded in acrylic resin inside plastic cylinders. A circular area (minimum diameter 3.6 mm) of enamel was exposed and polished with a polishing machine (LaboPol-21, Struers A/S, DK-2750 Ballerup, Denmark), using first a 180-grit (FEPA) and then a 2400-grit SiC paper. The coarser paper was used to remove a bulk of enamel to get a wide enough surface for the bonding, and the finer paper to finish and smooth the rough surface. A control group of intact enamel (*n* = 10) was also prepared, where the teeth were half-embedded in acrylic resin.

The enamel substrates were etched using a 32% phosphoric acid etching gel (Table 2) and a small cylindrical amount (height 2-3 mm, Ø 3.6 mm) of Transbond XT orthodontic adhesive (Table 2) was bonded on the enamel. The etching and bonding proceeded as follows: enamel was etched for 15 seconds, rinsed with water, and air-dried according to the manufacturer's instructions. A cylindrical (height 5 mm, Ø  3.6 mm) mold cut from a plastic tube was placed on the enamel, the adhesive was dispensed into the mold and light cured for 60 s (20 s from above and 20 s from two sides) with a handheld light-curing unit (light-emitting diode, Elipar S10, 3M ESPE, St. Paul, MN, USA), with the intensity of 1834.8 mW/cm^2^ and the wavelength peak of 455 nm ± 10 nm according to the manufacturer. The plastic mold was removed and the specimens were stored in distilled water in 37°C for four hours. After that the specimens were tested for initial bond strength with a testing machine (LLOYD Instruments, AMETEK Lloyd Instruments Ltd., West Sussex UK) with so-called shear bond strength test with cross-head speed of 1 mm/min. Load-displacement curves were recorded. Testing was made in air at room temperature. Then the specimens were stored overnight in distilled water in 37°C. The teeth in the control group of intact enamel were cleaned with pumice, rinsed with water, air-dried, and then etched, bonded, and tested with the same procedures as the rest of the specimens.

The next day the specimens were divided into four groups (*n* = 10): one group was left untouched and in three groups a small amount of enamel was ground off with an automatic polishing machine (RotoPol-11, Struers A/S, Pederstrupvej 84, DK-2750 Ballerup, Denmark) using a 4000-grit SiC paper (Struers A/SDK-2750 Ballerup, Denmark). The groups underwent grinding with the same settings (4000-grit SiC paper, 150 RPM, 5 N) but with different grinding times: 10 s, 20 s, and 30 s. The groups and their treatments are presented in Table 1. The roughness of the SiC papers was chosen so that with the other grinding factors they removed a desired amount of enamel. Then all the enamel substrates were etched and bonded again with the previously described procedures, stored for four hours, and tested for bond strength with LLOYD testing machine. The amount of enamel that was ground off was determined with additional measuring-samples: five substrates were each ground for 10, 20, and 30 seconds and measured. The samples were measured with a micrometer (Coolant Proof Micrometer, Mitutoyo Corporation, Japan). Every sample was measured five times to avoid error, and an average thickness was calculated for every sample. The average amount of enamel that was removed was 7 *µ*m (±2) for 10 s, 12 *µ*m (±1) for 20 s, and 16 *µ*m (±3) for 30 s. All procedures were performed by the same operator.

The enamel surfaces were also imaged for visual analysis using a scanning electron microscope (SEM, JSM-5500, Jeol USA, Inc., Peabody, MA). The substrates were gold-sputtered and imaged. A few substrates of interest of different treatments were selected for examining from the prepared groups. One sample was cut and imaged in transverse section, to observe the depth of the resin tags.

Surface roughness of one specimen from each group was determined by optical noncontacting profiler (Contour-GT-K1, Bruker, Billerica, MA, USA) and analysed with a Bruker Vision 64 software (version 5.41, update 4, Bruker, Billerica, MA, USA), to see how it would correspond to the view of the SEM micrographs. Microroughness of the surface was reported as average surface roughness (*R*_*a*_).

Statistical analysis was performed with SPSS Statistics version 22.0. The data was tested for normality and a one-way ANOVA was performed with a Tukey's post hoc test. Regression analysis was used to demonstrate correlation between grinding time, that is, removal of enamel layer before rebonding and the rebonding strength.

## 3. Results

### 3.1. Bond Strength Measurement

**Figure 1:**
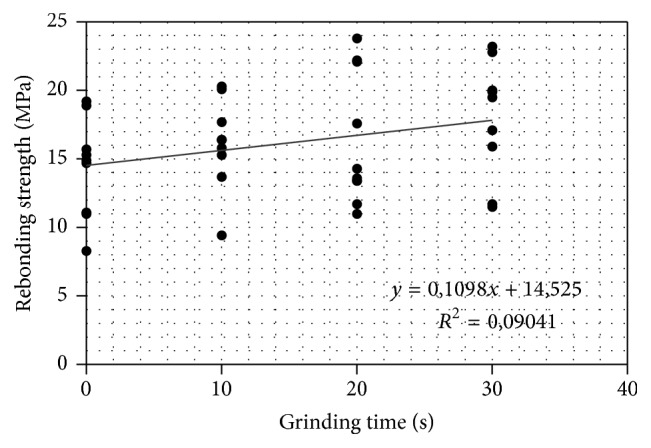
Regression line between the grinding time of enamel before rebonding and rebonding strength.

**Figure 2:**
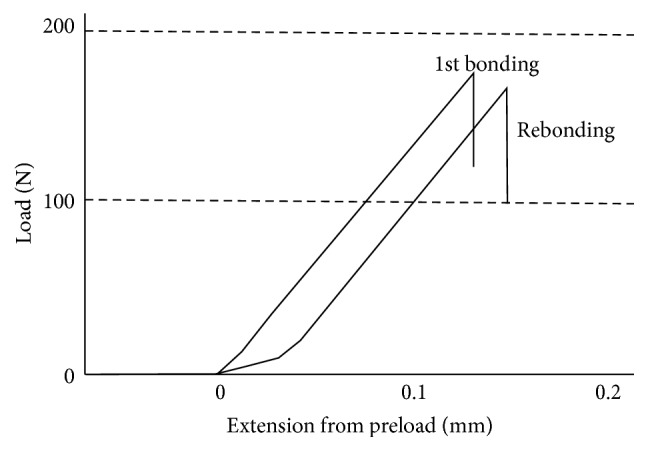
Typical load-extension curves of debonding the composite after the first bonding and after rebonding (curves are from groups G0 1st bonding and G0 rebonding).

The increase in the grinding time, that is, removal of the enamel layer, showed trend for higher rebonding strengths as demonstrated by the regression analysis (Figure 1). The mean bond strength value for intact enamel was 18.3 MPa (±3.4), and the bond strengths of the 1st bonding were 19.4 MPa (±5.2) for G0 group, 15.7 MPa (±5.2) for G1, 17.5 MPa (±4.9) for G2, and 20.3 MPa (±5.9) for G3. The rebonding strength for the G0 group was 14.3 MPa (±3.6), and the rebonding strengths for the ground substrate groups were 16.1 MPa (±3.3) for G1, 16.3 MPa (±4.8) for G2, and 18.0 MPa (±4.3) for G3 (Table 3). Although there was trend between the increase of rebonding strength and longer grinding time before rebonding (Figure 1), ANOVA did not show statistical differences between groups (*p* > 0.05). Within the groups, the values between first bonding and rebonding strengths differed significantly in G0 group (*p* < 0.05).  Figure 2 shows typical load-displacement curves demonstrating brittle type of debonding failure for the first bonding and minor ductility for the early stage of loading of the rebonded specimens. Grinding times, amounts of enamel loss, surface roughness parameters, and bond strengths are presented in Table 3. Average surface roughness (*R*_*a*_) for the intact enamel was 0.954 *μ*m, for the etched enamel (group E) 2.307 *μ*m, for group G0 0.301 *μ*m, for group G1 1.945 *μ*m, for group G2 0.857, and for group G3 0.343 (Table 4).

### 3.2. SEM Examination

**Figure 4:**
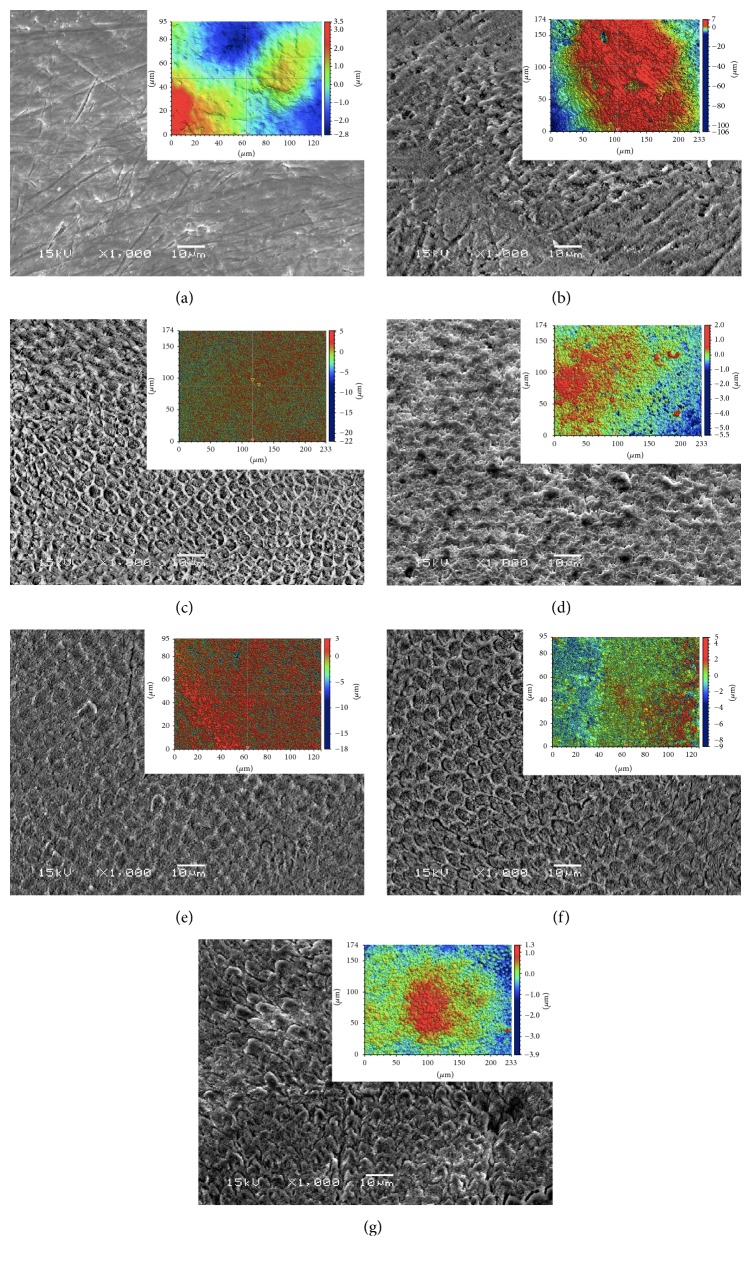
SEM images and corresponding optical profilometer image of the enamel surface of (a) intact, unetched enamel specimen (b) group E before bonding, (c) prepared enamel specimen before 1st bonding, (d) G0 before rebonding, (e) group G1 before rebonding, (f) group G2 before rebonding, and (g) group G3 before rebonding. Original magnification ×1000, bar = 10 *μ*m.

**Figure 5:**
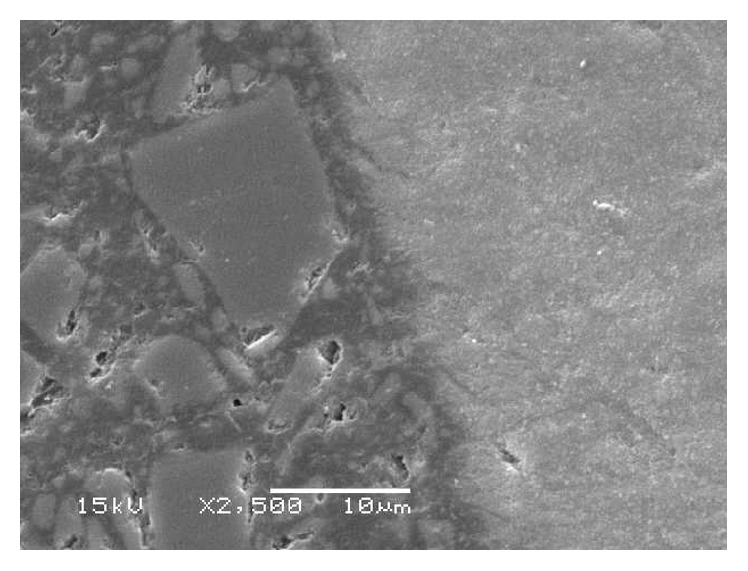
SEM image of the cross section of the interface between etched enamel and resin composite (original magnification ×2500, bar = 10 *μ*m).

Figure 4 shows representative SEM images and noncontacting profilometer images of the enamel surface of the study groups. The intact enamel surface exhibited signs of wear, that is, pits and grooves. The ground enamel surface was rather smooth, and grinding the enamel surface after the first bonding resulted in a similar surface as in the initial substrate surface, indicating that the adhesive was removed by the procedure. The reetched substrate surfaces showed clear etching patterns, though with different pattern types. The G0 substrate that was not ground before reetching and rebonding contained remnants of adhesive resin after reetching, whereas the ground substrates showed clearly etched enamel surfaces. It can be seen that the reetching of the not-ground substrate did not remove the remaining adhesive resin layer, but turned it into “adhesive resin-mash.” The most common fracture pattern, presented in almost all the specimens, was adhesive failure. The enamel fractured in three specimens. SEM of the cross section of the adhesive interface showed depth of the resin tags to be 5–10 *µ*m into the enamel (Figure 5).

## 4. Discussion

This study aimed to examine differences between removal of different amounts of enamel before rebonding. There was a trend indicating that higher rebonding strength was obtained with increasing enamel removal, although statistical analysis did not show strong relationship between the variables. It is likely that bigger number of specimens would have increased the statistical significance between the variables. It is also possible that the testing method to measure the bond strength with predominantly shear type of stress could have also contained microlocations of tensile stress which could have increased the standard deviations and lowered the statistical differences between the groups. The fracture type was brittle fracture as demonstrated by the load-extension curve (Figure 2). Brittleness of the fracture type is due to the cross-linked polymer matrix of the resin composite. Interestingly, the load-extension curve of the rebonded group showed at the early stage of the loading ductility of some degree which may relate to the presence of partly loose remnants of the resin composite and adhesive resin on the enamel surface on to which the rebonding was made. In the loading event, partly loose particles debond with lower level of stress which can be seen in early stage on the load-extension curve.

It was observed that the G0 group showed significantly higher initial bonding strength (19.4 MPa) than corresponding rebonding strength (14.3 MPa) which suggests that remnants of adhesive resin harm etching the surface for rebonding. Therefore, it is beneficial to reveal a fresh enamel surface before rebonding by grinding the surface. The intact enamel surface etched relatively well, despite presence of aprismatic layer or hypermineralization of the enamel surface.

The results of this study revealed that the differences between rebonding directly on the debonded surface or on a slightly ground surface were minor. If the rebonding strength is desired to be as high as the initial bonding strength, then removal of enamel can be recommended. A thickness of a maximum 17 *µ*m of enamel was removed during the grinding process resulting in a similar bond strength as with initial bonding of intact enamel. According to the literature, residual adhesive resin clean-up removes approximately 5–30 *µ*m of enamel [25–27], depending on the grinding system that is used. Even a loss of 40–60 *µ*m of enamel has been reported to occur in the entire debonding and clean-up procedure [6, 28]. In the present study, a 7 *µ*m grinding created a reetchable enamel surface which was also confirmed by the surface roughness measurement for *R*_*a*_ and *R*_*t*_. Interestingly, surface roughness parameters lowered when the enamel was ground further.

Grinding of enamel can be made clinically by rotating silicon carbide finishing bur and damage to the enamel is only minor [16, 29, 30]. On the other hand, there are reports stating that even with a clean-up with a silicon carbide finishing bur small remnants of adhesive can remain on enamel [19, 20]. Although it was found in the present and other studies [29] that the reetching produced a regular etching pattern to ground enamel surface after debonding, it has also been suggested that the reetching step could be omitted, to avoid risk of enamel fracture due to rebonding strengths that are too high [31].

Clinically, the desired bond strength is different in different areas of dentistry: in restorative dentistry the highest possible bond strength is desirable, whereas in orthodontics the bond must be strong enough to keep the appliances in place for the duration of the treatment but at the same time allow easy detachment of the appliance once the treatment is over. If the bond strength is too high, it can result in enamel damage or discomfort for the patient at the removal. Further research is needed to investigate the amount of enamel loss in debonding and rebonding process with brackets of different types and materials.

## 5. Conclusions

- Rebonding strengths without grinding the surface before bonding were lower than bond strength to intact enamel.
- Removal of small amount of enamel refreshed the surface for rebonding.

## Figures and Tables

**Figure 3:**
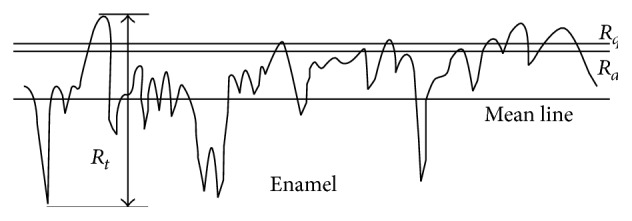
Description of surface roughness parameters: average roughness value (*R*_*a*_): the arithmetic mean of the height of peaks and depth of the valleys from a mean line (*R*_*t*_).

**Table 1:** Different groups and their treatments.

Group	Treatment
E	Intact enamel, etched, and bonded
G0	Prepared flat surface, etched, bonded and debonded, then reetched, and rebonded
G1	Prepared flat surface, etched, bonded and debonded, ground for 10 s, then reetched, and rebonded
G2	Prepared flat surface, etched, bonded and debonded, ground for 20 s, then reetched, and rebonded
G3	Prepared flat surface, etched, bonded and debonded, ground for 30 s, then reetched, and rebonded

**Table 2:** Materials used in the study.

Materials	Manufacturer	Lot no.	Contents	Wt%
Transbond XT	3M Unitek (Monrovia, CA, USA)	N568393	Silane treated quartz	70–80
			Bis-GMA	10–20
			EBPADMA	5–10
			Silane treated silica	<2

Etching gel	3M Unitek (Monrovia, CA, USA)	576331	Water	55–65^*∗*^
			Phosphoric acid	30–40^*∗*^
			Amorphous silica	5–10^*∗*^

Bis-GMA indicates bisphenol-A-diglycidyl ether dimethacrylate and EBPADMA bisphenol-A-bis(2-hydroxyethyl ether) dimethacrylate.

^*∗*^The specific chemical identity and/or exact percentage (concentration) of this composition has been withheld as a trade secret.

**Table 3:** Bond strengths (MPa) of the composite to enamel after the first bonding and rebonding. Enamel substrate has been ground for 0, 10, 20, and 30 seconds (s) and the corresponding removal of enamel is given in micrometers (*μ*m). Surface roughness after acid etching of the ground enamel substrate is given as value of average surface roughness (*R*_*a*_).

	G0	G1	G2	G3
Grinding time	0	10	20	30
Thickness of removed enamel	—	7 (±2)	12 (±1)	16 (±3)
Surface roughness (one specimen)	0.301	1.945	0.857	0.343
Bond strength (1st bonding)	19.4^*∗*^ (±5.2)	15.7 (±5.2)	17.5 (±4.9)	20.3 (±5.9)
Bond strength (rebonding)	14.3^*∗*^ (±3.6)	16.1 (±3.3)	16.3 (±4.8)	18.0 (±4.3)

An asterisk *∗* indicates statistical difference (*p* < 0.05) between values.

**Table 4:** Surface roughness parameters of *R*_*a*_ and *R*_*t*_ of the substrates of test groups in *μ*m. For defining parameters *R*_*a*_ and *R*_*t*_, see Figure 3.

	Intact enamel	Etched intact enamel (E)	Etched enamel before 1st bonding	Reetched after debonding (G0)	Ground 10 s, etched (G1)	Ground 20 s, etched (G2)	Ground 30 s, etched (G3)
*R*_*a*_	0.954	2.307	1.928	0.301	1.945	0.857	0.343
*R*_*t*_	6.245	113.359	27.084	7.463	21.344	13.688	5.175
